# FGF21 Mimics a Fasting-Induced Metabolic State and Increases Appetite in Zebrafish

**DOI:** 10.1038/s41598-020-63726-w

**Published:** 2020-04-24

**Authors:** Ayelén Melisa Blanco, Juan Ignacio Bertucci, Suraj Unniappan

**Affiliations:** 1 Laboratory of Integrative Neuroendocrinology, Department of Veterinary Biomedical Sciences, Western College of Veterinary Medicine, University of Saskatchewan, Saskatoon, Saskatchewan Canada; 2 Laboratorio de Fisioloxía Animal, Departamento de Bioloxía Funcional e Ciencias da Saúde, Facultade de Bioloxía and Centro de Investigación Mariña, Universidade de Vigo, Vigo Pontevedra, Spain; 3 Toxicology Centre, University of Saskatchewan, Saskatoon, Saskatchewan Canada

**Keywords:** Physiology, Systems biology

## Abstract

Fibroblast growth factor 21 (FGF21) is a member of the FGF superfamily that acts in an endocrine manner. FGF21 is a key regulator of energy balance and metabolism in mammals, and has emerged as a therapeutic potential for treating obesity and diabetes. Here, we report that mRNAs encoding FGF21 and its receptors are widely distributed within the zebrafish tissues and are importantly modulated by fasting (decreased in brain and liver, and increased in gut). FGF21 stimulates food intake in zebrafish, likely in part by modulating brain *npy/agrp* and *nucb2/nesfatin-1* and gut *ghrelin* and *cck* mRNA expression. In accordance with this orexigenic role, the expression of FGF21 and its receptors were observed to increase preprandially and decrease post-feeding in the foregut and/or liver. Finally, we found important evidence in favor of a role for FGF21 in regulating glucose and lipid metabolism in the zebrafish liver in a way that mimics a fasting metabolic state.

## Introduction

Fibroblast growth factors (FGFs) are a superfamily of proteins with mainly paracrine actions, among which FGF19, FGF21, and FGF23 are distinct due to the presence of a unique heparin-binding region. Because of this structural difference, these three family members do not adopt the conventional conformation of FGFs, which results in their ability to function as endocrine factors^1^. FGF21 was first identified in the mouse embryos as a 210-amino acid protein most abundantly expressed in the liver^2^. However, other tissues, including the thymus, white and brown adipose tissue, skeletal muscle, pancreas, gastrointestinal tract and the brain were described as production sites of FGF21 in rodents and human at a much lower extent^3,4^. FGF21 activates members of the FGF receptor (FGFR) family of receptor tyrosine kinases^5^, of which seven subtypes (1b, 1c, 2b, 2c, 3b, 3c, 4) with a tissue-specific expression have been identified in mammals^6^. The synthesis and release of FGF21 occur predominantly in response to different nutritional conditions (mainly fasting or specific diets^7^), although other factors, such as exercise, cold, hormones or the circadian system, have been also shown to regulate the synthesis of FGF21^8^.

FGF21 has been widely studied in mammals because of its key role in the regulation of energy balance, and carbohydrate and lipid metabolism^8–10^, specially in response to fasting. FGF21 increases gluconeogenesis and tricarboxylic acid cycle flux, stimulates hepatic fatty acid oxidation and ketogenesis, and suppresses hepatic *de novo* lipogenesis^11^ in the fasting state, or under conditions that mimick a fasting state^11–13^. Additionally, the administration of FGF21 to obese and diabetic mice reduces body weight, whole-body fat mass and liver triglyceride content, increases fat utilization and energy expenditure, and improves glucose tolerance and insulin sensitivity in the liver and adipose tissue^14–19^, placing FGF21 as a therapeutic potential for treating obesity and diabetes. Besides its major metabolic role, FGF21 has been shown to increase food and water intake^20,21^, induce anxiogenic behavior^22^, extend lifespan^23^, regulate muscle development^24^, and play a role in reproduction^25^ in mammals.

Only two studies are available on FGF21 in fish. One reported the *fgf21* cDNA sequence of zebrafish (*Danio rerio*) and described that FGF21 is essential for haematopoiesis in this species^26^. The second one identified the Asian seabass (*Lates calcarifer*) *fgf21* cDNA sequence and reported the expression of the gene to be restricted to the intestine and kidney, which differs from the mammalian expression profile^27^. Unlike in mammals, fasting downregulates *fgf21* mRNAs in the intestine and kidney and that administration of FGF21 suppresses appetite in the Asian seabass. Given the great importance of FGF21 in regulating metabolic processes in mammals, we aimed to study the regulation of FGF21 by feeding and nutritional status, and to determine whether FGF21 modulates food intake and glucose and lipid metabolism in zebrafish. The specific objectives were to: (i) describe the distribution of mRNAs encoding FGF21 and its receptors in tissues involved in feeding and metabolism in zebrafish, (ii) determine the action of FGF21 on zebrafish food intake, (iii) study the periprandial profiles and fasting-induced changes in the expression of the FGF21 system (namely, FGF21 and its receptors), (iv) study the putative role of FGF21 on the expression and activity of enzymes related to glucose and lipid metabolism *in vivo* and *in vitro*, and (v) determine whether the key transcription factors and coactivators mediating FGF21 actions in mammals (mainly peroxisome proliferator-activated receptor alpha, PPARα, and peroxisome proliferator-activated receptor gamma coactivator 1-alpha,PPARGC1α) may also operate in zebrafish.

## Results

### FGF21 and its receptors are expressed in tissues involved in feeding and metabolism in the zebrafish

**Figure 1:**
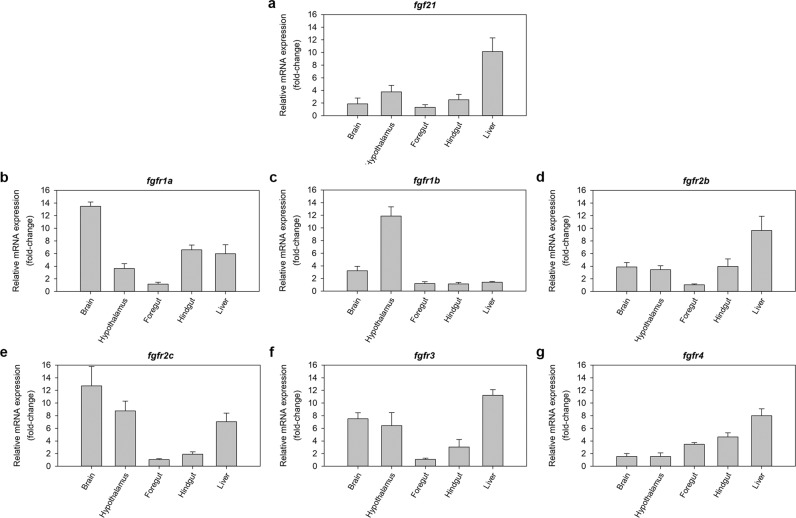
Distribution of mRNAs encoding FGF21 and its receptors in tissues involved in feeding and metabolism in zebrafish. Quantitative analysis of mRNA expression was performed by RT-qPCR considering *β-actin* as reference gene. Data are expressed as mean + SEM (n = 6), relative to the tissue with the lowest mRNA expression. *fgf21*, fibroblast growth factor 21; *fgfr*, fibroblast growth factor receptor.

The distribution of mRNAs encoding FGF21 and its receptors in zebrafish tissues involved in feeding and metabolism is shown in Fig. 1. Expression of *fgf21* and all receptors studied (*fgfr1a*, *fgfr1b*, *fgfr2b*, *fgfr2c*, *fgfr3* and *fgfr4*) was detected in the brain, hypothalamus, foregut, hindgut and liver. Among them, *fgf21*, *fgfr2b*, *fgfr3* and *fgfr4* mRNAs were most abundant in the liver compared to other tissues, while *fgfr1a*, *fgfr1b* and *fgfr2c* mRNAs were more abundant in the brain and/or hypothalamus.

### FGF21 increases food intake in zebrafish

**Figure 2:**
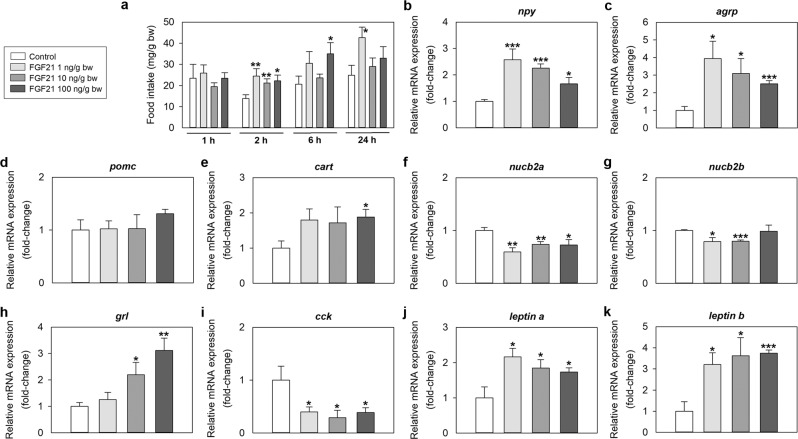
Effects of FGF21 on feeding regulation in zebrafish. (**a**) Food intake in zebrafish 1, 2, 6 and 24 h after intraperitoneal administration of saline alone (control) or containing 1, 10 or 100 ng/g bw of FGF21. Results correspond to the mean + SEM of the results obtained in three different experiments (n = 3 in each experiment). Asterisks denote significant differences between control and treated groups assessed by t-test (*p < 0.05, **p < 0.01). **(b–k)** Expression of mRNAs encoding key appetite-regulating peptides in the zebrafish brain (**b–g**), gut (**h–i**) and liver (**j–k**) 2 h after intraperitoneal administration of saline alone (control) or containing 1, 10 or 100 ng/g bw of FGF21. mRNA expression was quantified by RT-qPCR considering *β-actin* as reference gene. Data are expressed as mean + SEM (n = 6). Asterisks denote significant differences between control and treated groups (*p < 0.05, **p < 0.01, ***p < 0.001). *agrp*, agouti-related protein; *cart*, cocaine- and amphetamine-regulated transcript; *cck*, cholecystokinin; FGF21, fibroblast growth factor 21; *grl*, ghrelin; *npy*, neuropeptide Y; *nucb2*, nucleobindin 2; *pomc*, proopiomelanocortin.

Intraperitoneal (IP) administration of human recombinant FGF21 resulted in a significant increase in food intake at 2 h (all doses tested), 6 h (only 100 ng/g bw dose) and 24 h (only 1 ng/g bw dose) post-injection when compared to the control groups. Magnitude of feeding increase was about 60–80% in all cases. No significant differences in food intake were observed between saline and FGF21-injected fish at 1 h post-injection (Fig. 2a). Injection of all doses of FGF21 also caused a significant upregulation of *neuropeptide y* (*npy*) and *agouti-related protein* (*agrp*) mRNAs, and a significant reduction in *nucleobindin 2a* (*nucb2a*) and *nucleobindin 2b* (*nucb2b*) mRNAs in the zebrafish brain (Fig. 2b,c,f and g). Brain levels of *cocaine- and amphetamine-regulated transcript* (*cart*) mRNAs were slightly inducted (0.8-fold) by the peptide, but only at the highest dose (Fig. 2e). In peripheral tissues, IP injection of FGF21 downregulated the expression of *cholecystokinin* (*cck*) mRNAs in the foregut (Fig. 2i), and caused a significant increase in mRNAs encoding ghrelin in the foregut (Fig. 2h), and leptin a and leptin b in the liver (Fig. 2j,k).

### Fasting modulates the mRNA expression of the FGF21 system in zebrafish

**Figure 3:**
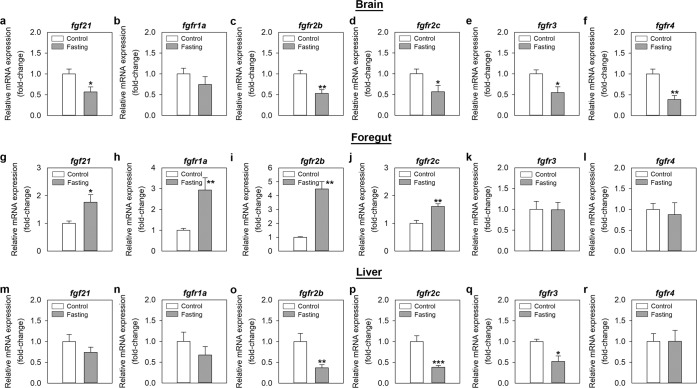
Effects of 7-day fasting on the mRNA expression of *fgf21* and its receptors in the zebrafish brain (**a–f**), foregut (**g–l**) and liver (**m–r**). Data obtained by RT-qPCR are expressed as mean + SEM (n = 6). Asterisks denote significant differences between control and treated groups (*p < 0.05, **p < 0.01, ***p < 0.001). *fgf21*, fibroblast growth factor 21; *fgfr*, fibroblast growth factor receptor.

A 7-day food deprivation significantly reduced FGF21 and the FGF21 receptor subtypes 2b, 2c, 3 and 4 mRNAs in the zebrafish brain (Fig. 3a–f). On the contrary, fasting upregulated the mRNA expression of *fgf21* (≈2-fold), *fgfr1a* (3-fold), *fgfr2b* (4.5-fold) and *fgfr2c* (≈1.5-fold) in the foregut (Fig. 3g–j). No fasting-induced changes were observed for *fgfr3* and *fgfr4* in this tissue (Fig. 3k,l). In the liver, fish that were food deprived during 7 days showed significantly lower levels of *fgfr2b*, *fgfr2c* and *fgfr3* mRNAs when compared to control fed fish (Fig. 3o–q). Hepatic mRNA levels of *fgf21, fgfr1a* and *fgfr4* were unaltered by fasting (Fig. 3m,n,r).

### The FGF21 system displays periprandial variations in expression in the zebrafish gut and liver

**Figure 4:**
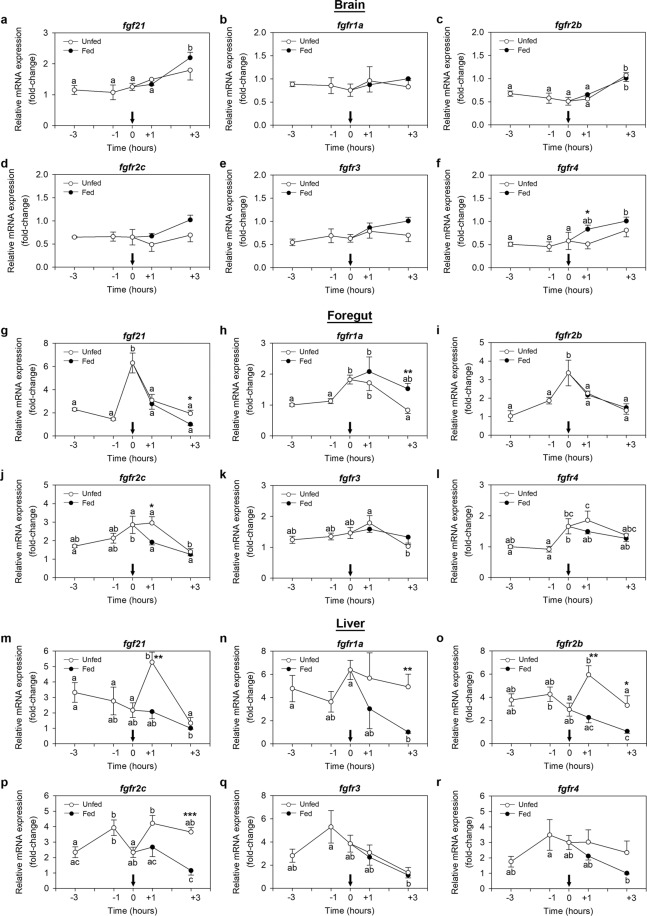
Periprandial changes in the mRNA expression of *fgf21* and its receptors in the zebrafish brain (**a–f**), foregut (**g–l**) and liver (**m–r**). Samples were collected before scheduled feeding time (−3 h and −1 h), at feeding time (0 h) and after scheduled feeding time (+1 h and +3 h) in both fed and unfed fish. Data are expressed as mean ± SEM (n = 6) relative to the lowest average expression. Arrows denote feeding time. Different letters indicate significant differences (p < 0.05) among the different time points in fed (black dots) or unfed (white dots) groups, while asterisks indicate significant differences (*p < 0.05, **p < 0.01, ***p < 0.001) between groups at the same time point. *fgf21*, fibroblast growth factor 21; *fgfr*, fibroblast growth factor receptor.

Figure 4 shows the periprandial mRNA expression profiles of *fgf21* and its receptors in the zebrafish brain, foregut and liver. Major findings described a significant preprandial increase in the expression of *fgf21*, *fgfr1a*, *fgfr2b* and *fgfr4* in the foregut, as demonstrated by significant higher levels of mRNAs at scheduled feeding time (0 h) compared to levels at −1 h and −3 h (Fig. 4g–i,l). Expression of *fgf21* and *fgfr2b* decreased after feeding (+1 h and +3 h), although a similar drop was observed in unfed fish (Fig. 4g,i). *Fgfr1a* mRNA levels remained unaltered after feeding in the foregut of fish that received food; however, a significant drop was detected at +3 h compared to 0 h in those fish that skipped the scheduled feeding (Fig. 4h). No significant postprandial variations were observed in *fgfr4* expression (Fig. 4l). As for *fgfr2c* and *fgfr3*, mRNA levels did not change preprandially in the foregut, and only a postprandial reduction in expression was observed for *fgfr2c* in both fed and unfed fish and for *fgfr3* in unfed fish (Fig. 4j,k).

In the liver, almost no preprandial variations in the levels of mRNAs encoding FGF21 or any of its receptors were detected. Nevertheless, we observed that mRNA expression of almost all of the genes studied decreased in the liver after a meal in fish that were fed at their scheduled feeding time, while remained high in food deprived fish (Fig. 4m–r). The only exception to this profile was observed for *fgfr3* mRNAs, whose levels decreased at +3 h both in fed and unfed fish (Fig. 4q). In the brain, no major periprandial changes were observed for any of the genes studied. Only a slight increase in *fgf21*, *fgfr2b* and *fgfr4* mRNAs was detected at +3 h in fed fish (Fig. 4a–f).

### FGF21 modulates the expression of glucose transporters and genes involved in glucose metabolism in the zebrafish liver *in vivo* and *in vitro*

**Figure 5:**
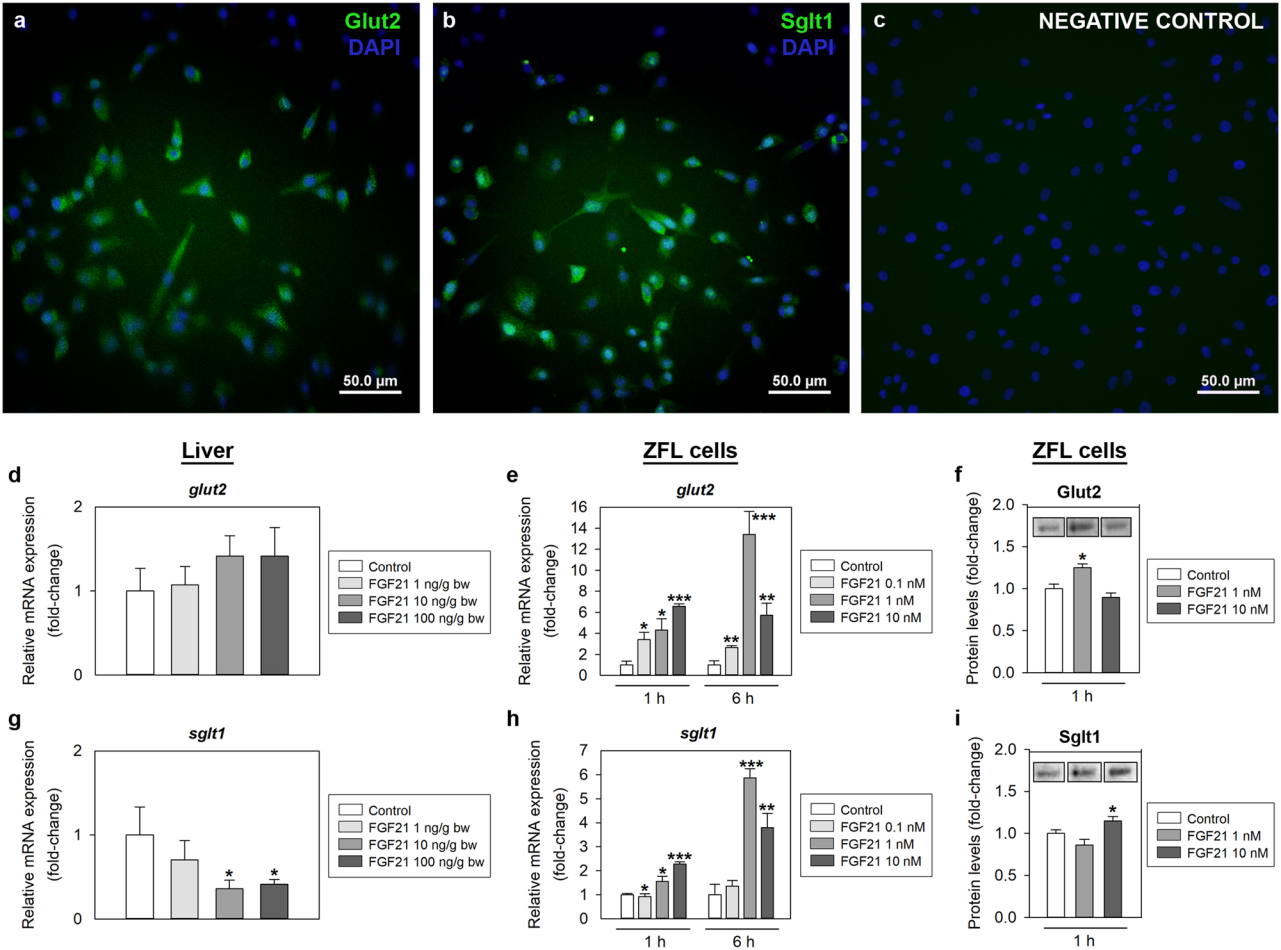
Effects of FGF21 on glucose transporters in zebrafish liver. (**a–c**) Glut2-like (a; green) and Sglt1-like (b; green) immunoreactivity in ZFL cells detected by immunohistochesmistry. Negative control (**c**) was incubated in the absence of primary antibody. All images are merged with DAPI showing nuclei in blue. Scale bars (µm) are indicated in each image. **(d,g)** Expression of mRNAs encoding Glut2 and Sglt1 in the zebrafish liver 1 h after intraperitoneal administration of saline alone (control) or containing 1, 10 or 100 ng/g bw of FGF21. Data obtained by RT-qPCR are expressed as mean + SEM (n = 6). Asterisks denote significant differences between control and treated groups assessed by t-test (*p < 0.05). **(e,h)** Concentration and time-dependent effects of FGF21 on glucose transporters gene expression in ZFL cells. Cells were incubated with culture media alone (control) or containing different concentrations of FGF21 (0.1, 1 and 10 nM) during 1 and 6 h. Data obtained by RT-qPCR are shown as mean + SEM of the results obtained in two different experiments (n = 6 in each experiment). Asterisks denote significant differences between control and treated groups (*p < 0.05, **p < 0.01, ***p < 0.001). **(f,i)** Protein levels of Glut2 and Sglt1 in ZFL cells 1 h after exposure to 1 or 10 nM FGF21. Data obtained by Western blot is shown as mean + SEM (n = 4). A representative cropped blot per treatment is shown. Full-length blots/gels are presented in Supplementary Figure 1. Asterisks denote significant differences between control and treated groups (*p < 0.05). FGF21, fibroblast growth factor 21; Glut2, glucose transporter 2; Sglt1, sodium-glucose cotransporter 1.

The effects of FGF21 on the expression of glucose transporters (Glut2, glucose transporter 2, and Sglt1, sodium-glucose cotransporter 1) in the zebrafish liver are shown in Fig. 5. IP administration of FGF21 did not modulate the mRNA levels of *glut2* at 2 h post-injection (Fig. 5d). However, it caused a significant reduction in the hepatic *sglt1* mRNA expression at doses of 10 and 100 ng/g bw (Fig. 5g). FGF21-induced changes in the expression of Glut2 and Sglt1 were also studied *in vitro* using ZFL cells. Prior to this, the location of the two transporters within such cells was described using immunocytochemistry. Both Glut2 and Sglt1 were abundant in the ZFL cells and located within the cytoplasm of the cells (Fig. 5a,b). Levels of *glut2* and *sglt1* mRNAs were upregulated in ZFL cells exposed to different concentrations of FGF21 (0.1, 1 and 10 nM) during 1 and 6 h (Fig. 5e,h). Likewise, the exposure of cells to 1 nM and 10 nM FGF21 for 1 h increased Glut2 and Sglt1 protein levels, respectively (Fig. 5f,i).

**Figure 6:**
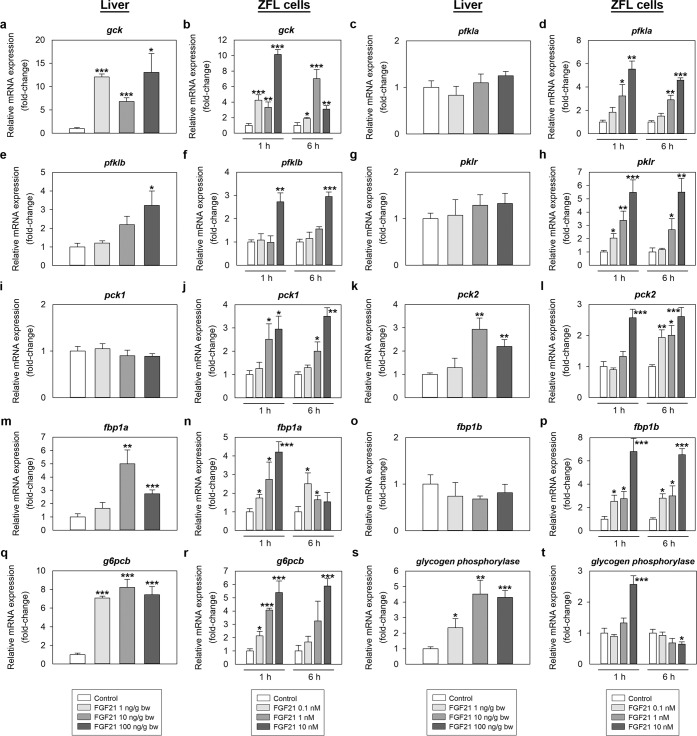
*In vivo* (first and third column) and *in vitro* (second and fourth column) effects of FGF21 on the mRNA expression of genes encoding key enzymes involved in glucose metabolism in zebrafish. (**a,c,e,g,i,k,m,o,q,s**) Expression of genes involved in glucose metabolism in the zebrafish liver 1 h after intraperitoneal administration of saline alone (control) or containing 1, 10 or 100 ng/g bw of FGF21. Data are expressed as mean + SEM (n = 6). Asterisks denote significant differences between control and treated groups assessed by t-test (*p < 0.05, **p < 0.01, ***p < 0.001). (b, d, f, h, j, l, n, p, r, t) Levels of mRNAs encoding key enzymes involved in glucose metabolism in ZFL cells exposed to 0.1, 1 and 10 nM FGF21 during 1 and 6 h. Data are shown as mean + SEM of the results obtained in two different experiments (n = 6 in each experiment). Asterisks denote significant differences between control and treated groups (*p < 0.05, **p < 0.01, ***p < 0.001). *fbp1a*, *fructose 1,6-bisphosphatase 1a*; *fbp1b*, *fructose 1,6-bisphosphatase 1b*; FGF21, fibroblast growth factor 21; *g6pcb*, *glucose 6-phosphatase b*; *gck*, *glucokinase*; *pck1*, *phosphoenolpyruvate carboxykinase 1*; *pck2*, *phosphoenolpyruvate carboxykinase 2*; *pfkla*, *phosphofructokinase a*; *pfklb*, *phosphofructokinase b*; *pklr*, *pyruvate kinase*.

Expression of genes involved in glucose metabolism was also modulated by treatment with FGF21, as shown in Fig. 6. When administered intraperitoneally, all doses of FGF21 tested (1, 10 and 100 ng/g bw) caused an induction in the levels of *glucokinase* (*gck*), *glucose 6-phosphatase b* (*g6pcb*) and *glycogen phosphorylase* mRNAs, while 10 and 100 ng/g bw FGF21 (but not 1 ng/g) upregulated the mRNA expression of *phosphoenolpyruvate carboxykinase 2* (*pck2*) and *fructose 1,6-bisphosphatase 1a* (*fbp1a*) at 2 h post-injection (Fig. 6a,k,q,m,s). We also observed an increase in *phosphofructokinase b* (*pfklb*) mRNAs upon administration of 100 ng/g bw FGF21 (Fig. 6e). No changes in the levels of *phosphofructokinase a* (*pfkla*), *pyruvate kinase* (*pklr*), *phosphoenolpyruvate carboxykinase 1* (*pck1*) and *fructose 1,6-bisphosphatase 1b* (*fbp1b*) mRNAs were observed following IP treatment with FGF21 (Fig. 6c,g,i,o). *In vitro* exposure of ZFL cells to FGF21 during 1 and 6 h resulted in a concentration-dependent induction in the expression of *gck*, *pfkla*, *pfklb*, *pklr*, *pck1*, *pck2*, *fbp1a*, *fbp1b* and *g6pcb* (Fig. 6b,d,f,h,j,l,n,p,r) mRNAs. *Glycogen phosphorylase* mRNAs were also upregulated by the exposure to 10 nM FGF21 during 1 h, but was found downregulated at 6 h (Fig. 6t).

### Genes implicated in lipid metabolism are regulated by FGF21 in the zebrafish liver *in vivo* and *in vitro*

**Figure 7:**
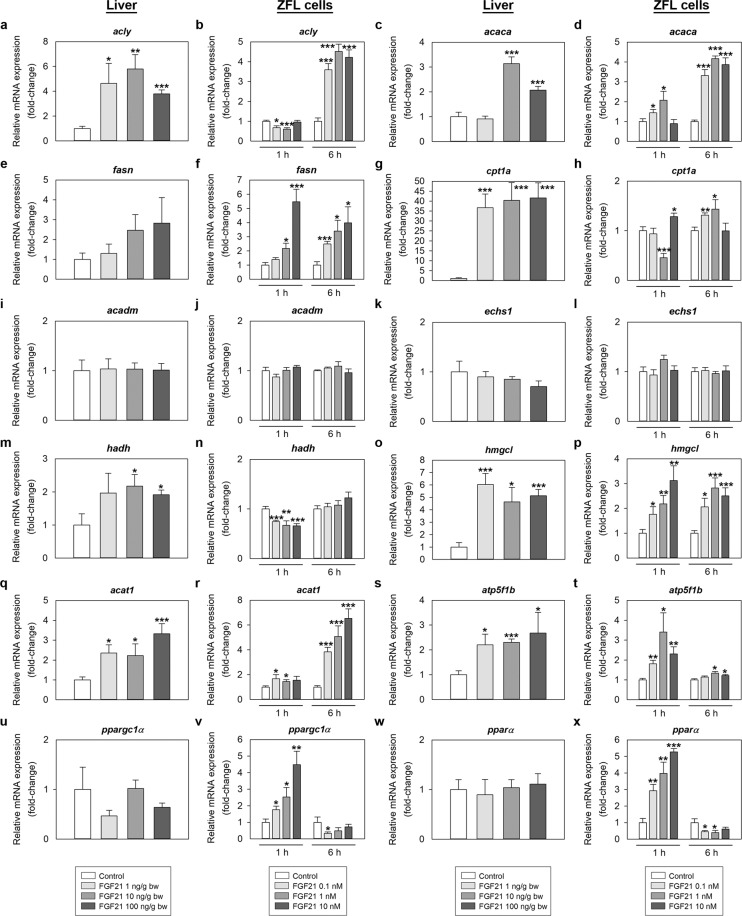
*In vivo* (first and third column) and *in vitro* (second and fourth column) effects of FGF21 on the mRNA expression of genes involved in lipid metabolism, mitochondrial activity and transcription factors in zebrafish. (**a,c,e,g,i,k,m,o,q,s,u,w**) Gene expression in the zebrafish liver 1 h after intraperitoneal administration of saline alone (control) or containing 1, 10 or 100 ng/g bw of FGF21. Data are expressed as mean + SEM (n = 6). Asterisks denote significant differences between control and treated groups assessed by t-test (*p < 0.05, **p < 0.01, ***p < 0.001). (**b,d,f,h,j,l,n,p,r,t,v,x**) Levels of mRNAs in ZFL cells exposed to 0.1, 1 and 10 nM FGF21 during 1 and 6 h. Data are shown as mean + SEM of the results obtained in two different experiments (n = 6 in each experiment). Asterisks denote significant differences between control and treated groups (*p < 0.05, **p < 0.01, ***p < 0.001). *acaca*, *acetyl-CoA carboxylase*; *acadm*, *acyl-CoA dehydrogenase medium-chain*; *acat1*, acetyl-CoA *acetyltransferase 1*; *acly*, *ATP citrate lyase*; *atp5f1b*, *ATP synthase F1 subunit beta*; *cpt1a*, *carnitine palmitoyltransferase 1a*; *echs1*, *enoyl-CoA hydratase short-chain 1*; *fasn*, *fatty acid synthase*; FGF21, fibroblast growth factor 21; *hmgcl*, 3-*hydroxy-3-methylglutaryl-CoA lyase*; *hadh*, *3-hydroxyacyl CoA dehydrogenase*; *pparα*, *peroxisome proliferator-activated receptor alpha*; *ppargc1α*, *peroxisome proliferator-activated receptor gamma coactivator 1-alpha*.

Figure 7 shows the FGF21-induced changes in the expression of genes involved in lipid metabolism in zebrafish liver. IP administration of 1, 10 and 100 ng/g bw FGF21 was observed to significantly upregulate the expression of *ATP citrate lyase* (*acly*), *carnitine palmitoyltransferase 1a* (*cpt1a*), *3-hydroxy-3-methylglutaryl-CoA lyase* (*hmgcl*) and *acetyl-CoA acetyltransferase 1* (*acat1*) mRNAs at 2 h post-injection (Fig. 7a,g,o,q). Levels of *acetyl-CoA carboxylase* (*acaca*) and *3-hydroxyacyl CoA dehydrogenase* (*hadh*) mRNAs were also increased by FGF21, but at doses of 10 and 100 ng/g (not 1 ng/g) (Fig. 7c,m). Injection of FGF21 did not alter the expression of *fatty acid synthase* (*fasn*), *acyl-CoA dehydrogenase medium-chain* (*acadm*) and *enoyl-CoA hydratase short-chain 1* (*echs1*) at the time tested (Fig. 7e,i,k). *In vitro*, exposure of ZFL cells to FGF21 dose-dependently induced levels of *acaca*, *fasn*, *cpt1a*, *hmgcl* and *acat1* mRNAs at 1 and 6 h, while reduced the expression of *hadh* at 1 h (Fig. 7d,f,h,p,r). *Acly* mRNA expression in ZFL cells were significantly reduced by treatment with FGF21 at 1 h, but increased at 6 h (Fig. 7b). No significant FGF21-induced changes in mRNA expression were observed for *acadm* and *echs1 in vitro* (Fig. 7j,l).

### FGF21 affects the expression of genes regulating mitochondrial activity and metabolic transcription factors

Expression of *ATP synthase F1 subunit beta* (*atp5f1b*) mRNAs was observed to be upregulated by FGF21 both *in vivo* at 2 h post-IP injection, and *in vitro* 1 and 6 h after exposure of ZFL cells to the peptide (Fig. 7s,t). Expression of the transcription factor *pparα* and coactivator *ppargc1α* was unaltered by IP injection of FGF21 (Fig. 7u,w). However, *in vitro* exposure of ZFL cells to FGF21 resulted in a significant induction in *ppargc1α* and *pparα* mRNAs at 1 h, while a significant reduction was observed at 6 h (Fig. 7v,x).

### FGF21 regulates the activity of key enzymes involved in glucose and lipid metabolism *in vitro*

**Figure 8:**
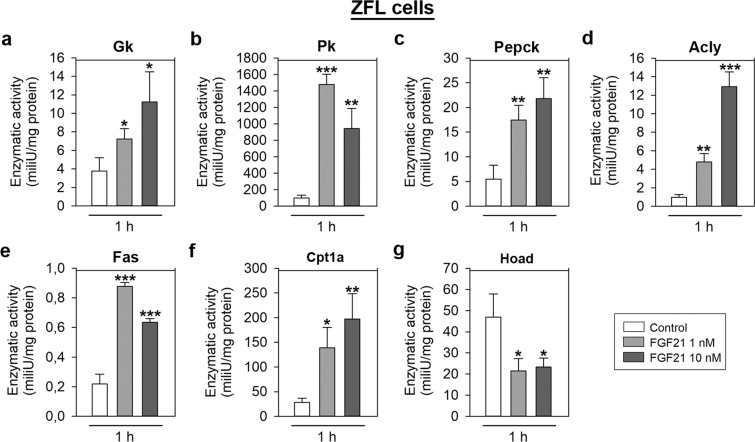
Effects of FGF21 on the activity of key enzymes involved in glucose and lipid metabolism in ZFL cells. Cells were incubated with culture media alone (control) or containing 1 or 10 nM FGF21 during 1 h. Data are shown as mean + SEM (n = 8). Asterisks denote significant differences between control and treated groups (*p < 0.05, **p < 0.01, ***p < 0.001). Acly, ATP citrate lyase; Cpt1a, carnitine palmitoyltransferase 1a; Fas, fatty acid synthase; FGF21, fibroblast growth factor 21; Gck, glucokinase; Hoad, 3-hydroxyacyl CoA dehydrogenase; Pepck, phosphoenolpyruvate carboxykinase; Pk, pyruvate kinase.

Exposure of ZFL cells to 1 and 10 nM FGF21 during 1 h resulted in a significant increase in the activity of Gck (≈2- and 3-fold), pyruvate kinase (Pk; ≈15- and 10-fold), phosphoenolpyruvate carboxykinase (Pepck; ≈3- and 4-fold), Acly (≈4- and 12-fold), fatty acid synthase (Fas; ≈4- and 3-fold) and Cpt1a (≈5- and 7-fold; Fig. 8a–f). Activity of 3-hydroxyacyl CoA dehydrogenase (Hoad) was decreased 2-fold in ZFL cells treated with FGF21 (Fig. 8g).

## Discussion

This research considered the nutritional regulation of the FGF21 system in zebrafish, and determined the putative role of this peptide in glucose and lipid metabolism for the first time in a non-mammal. First, we reported that *fgf21* mRNAs are present in zebrafish tissues involved in feeding and metabolism (brain, gut and liver), with considerably higher levels in the liver. Such abundant expression of FGF21 in the liver agrees with reports on tissue distribution in mammals^2,28^, but is not in agreement with the study by Wang and co-workers showing that *fgf21* mRNAs in the Asian seabass are exclusively expressed in the kidney and intestine^27^. As it will also be pointed out later, it seems that the Asian sea bass gene encoding FGF21 might have evolved differently compared to mammals (and potentially other fish species, including the zebrafish) and might exert different physiological functions in this species. We also observed a tissue-specific presence of mRNAs encoding FGF21 receptors in the zebrafish brain, gut and liver. Such differential expression of FGF21 receptor subtypes points to tissue specificity in mediating the different physiological actions that FGF21 might be exerting in zebrafish.

The presence of mRNAs encoding FGF21 and some of its receptors in the zebrafish brain and gut, key tissues involved in appetite regulation, suggests the involvement of FGF21 in feed intake control. Indeed, our results showed that human recombinant FGF21 increases short-term food intake in the zebrafish in a time- and dose-dependent manner when administered intraperitoneally. We used human peptide because, given the large length of FGF21, the synthesis of the corresponding fish peptide was prohibitive due to excessively high costs. While this is a limitation of the study, human recombinant FGF21 has been previously used to study feeding behavior in teleosts^27^. In addition, zebrafish FGF21 sequence maintains a conserved cysteine residue at position 122 of human *FGF21* protein. Both zebrafish and human FGF21 sequences share several residues at the C- and N-terminus (Supplementary Figure 2), which in mammals have been demonstrated to be critical for the interaction with the co-receptor b-Klotho and FGF receptors, respectively^29,30^. Additional studies are required to elucidate the structural characteristics of FGF21 and its receptor interactions, as well as mechanism of action in zebrafish. The observation of FGF21 being an orexigen in zebrafish is concordant with previous observations in rats intracerebroventricularly injected with FGF21^20^, although other studies reported no difference in food intake upon infusion of FGF21 in rats or in FGF21-KO mice^23^. However, it is opposite to the anorexigenic role reported for FGF21 in the Asian sea bass^27^, reinforcing the hypothesis of the FGF21 gene evolving independently in this species. The orexigenic role of FGF21 in zebrafish is likely mediated by the upregulation of orexigens NPY and AgRP in the brain and ghrelin in the foregut, and the downregulation of the anorexigens NUCB2/nesfatin-1 in the brain and CCK in the foregut. In fact, this is supported by our qPCR results, but further studies are needed to confirm this. Recinella and coworkers^20^ described that NPY and AgRP gene expression is increased, while expression of POMC and CART is decreased, in response to FGF21 in rats. Furthermore, FGF21-KO mice had higher levels of *Pomc* while lower levels of *Agrp* mRNAs in the hypothalamus compared to wild-type mice^31^. To exert actions in the zebrafish brain, it is likely that FGF21 crosses the blood-brain barrier, as it has been demonstrated in mammals^32^. In accordance with the orexigenic role reported in this study for FGF21 in zebrafish, we observed that mRNAs encoding FGF21 and its receptors in the foregut rise preprandially and decrease after a meal. Likewise, although no preprandial variations were detected, mRNAs encoding all components of the FGF21 system were reduced by feeding in the liver. This is the first report describing the periprandial variations in the FGF21 system in any species. Besides the periprandial profiles, the increase in the foregut expression of *fgf21* and almost all of its receptors in response to fasting observed in this study is also a clear signal of an appetite enhancer. However, such a response was not observed in the brain and liver. Instead, our results demonstrated that a 7-day fasting significantly reduces the expression of FGF21 system mRNAs in zebrafish tissues. These observations contradicted with that of studies in mammals^12,33–35^, and might not be related to the role of FGF21 on zebrafish food intake, but with a different physiological action considering the multifunctional nature of the peptide suggested by its wide tissue distribution in this species. Differences in study methods, including the species used, time of sampling and the regions of tissues selected will all contribute to the difficulties in direct comparisons between this research and other studies. Further studies are needed to elucidate the physiological meaning of the fasting-induced downregulation of the FGF21 system in the zebrafish brain and liver. In addition, future research should consider studying long-term effects of FGF21 on feeding and body weight, as well as muscle and fat mass in fish.

**Figure 9:**
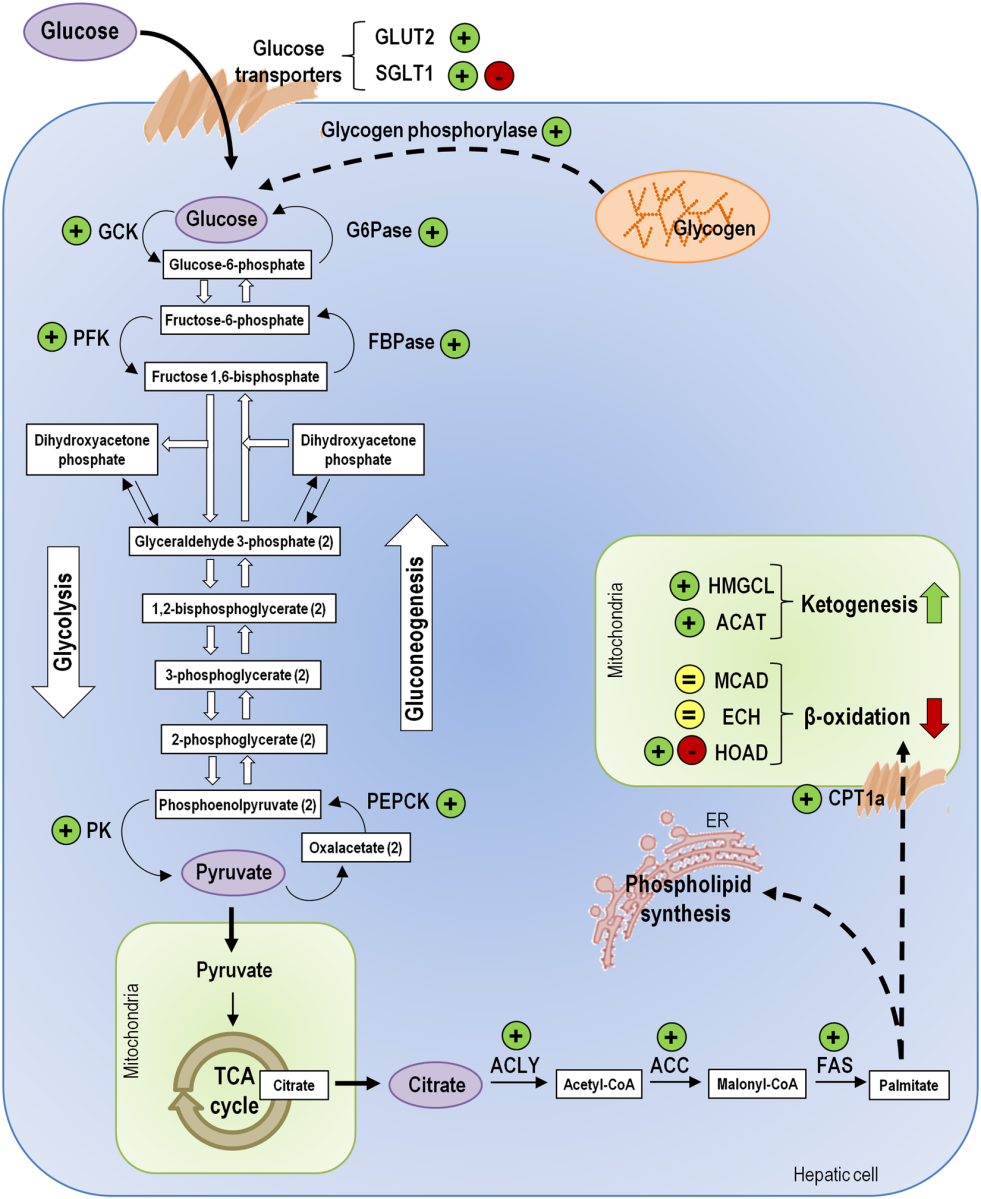
Schematic representation of the metabolic pathways proposed to be modulated by FGF21 in the zebrafish hepatic cells. Diagram shows the main pathways studied, and the transporters and enzymes whose mRNA expression (and activity for some of them) was shown to be upregulated (+), downregulated (−) or unaltered (=) by FGF21 treatment *in vivo* and/or *in vitro*. Based on the results of the present study, we hypothesize that FGF21 leads to an increase in the amount of glucose in the hepatic cells by enhancing its entrance into the cells, gluconeogenesis and glycogen degradation. Interestingly, we also observed evidence in favor of FGF21 stimulating not only gluconeogenesis but also glycolysis. We hypothesize that the final product of glycolysis, pyruvate, after being converted into citrate, might be released into the cytoplasm and be used as a substrate for fatty acid biosynthesis. Our results do not support that the increase in fatty acid synthesis is related to its β-oxidation, but instead it might be related to the synthesis of phospholipids for maintaining cell membrane structure. In addition, we propose that FGF21 might stimulate ketogenesis. ACAT, Acetyl-CoA acyltransferase; ACC, Acetyl-CoA carboxylase; ACLY, ATP citrate lyase; CPT1a, Carnitine palmitoyltransferase 1a; ECH, Enoyl-CoA hydratase; ER, endoplasmic reticulum; FAS, Fatty acid synthase; FBPase, Fructose 1,6-bisphosphatase; G6Pase, Glucose 6-phosphatase; GCK, Glucokinase; GLUT2, Glucose transporter 2; HMGCL, 3-hydroxy-3-methylglutaryl-CoA lyase; HOAD, 3-hydroxyacyl-CoA dehydrogenase; MCAD, Medium-chain acyl-coenzyme A dehydrogenase; PEPCK, Phosphoenolpyruvate carboxykinase; PFK, Phosphofructokinase; PK, Pyruvate kinase; SGLT1, sodium-glucose cotransporter 1; TCA cycle, tricarboxylic acid cycle (Krebs cycle).

Given the great abundance of the FGF21 system in the zebrafish liver, we hypothesized that FGF21 might also have an important role in glucose and lipid metabolism in zebrafish. Such a role for FGF21 has been reported in mammals (see reviews^10,36^), but not in fish. Our results demonstrated that FGF21 regulates genes and enzymes involved in glucose and lipid metabolism in the zebrafish liver (Fig. 9), in a way that metabolic changes expected by results observed would mimic the metabolic effects of fasting, as in mammals^13^. Various observations support this major finding. First, FGF21 was found to upregulate the mRNA and protein levels of glucose transporters Glut2 and Sglt1 *in vitro*, which might suggest an increase in the amount of glucose entrance into hepatocytes. The reduced expression of *sglt1* after FGF21 IP administration does not match the *in vitro* observations. This might be related to the fact that Sglt1 is an active transporter, and it might not be beneficial to use energy to increase the rate of glucose entrance in an *in vivo* situation. The suggested increase in the amount of glucose in the hepatic cells in response to FGF21 is also supported by the increased activity and/or expression of mRNAs encoding the gluconeogenic enzymes Pepck, fructose 1,6-bisphosphatase (Fbpase) and glucose 6-phosphatase (G6pase), and the increased mRNA levels of *glycogen phosphorylase* (implicated in glycogen degradation). In mammals, a FGF21-induced increase in gluconeogenesis and in the mRNA expression of *G6pc* and *Pck* was also described^13,37^. Previous reports have described that the expression of hepatic gluconeogenic enzymes^38^ and glycogenolysis^39^ are induced by leptin in fish. The increase in *leptin a* and *leptin b* mRNAs in the zebrafish liver after IP administration of FGF21 observed in this study suggests that the putative stimulation of gluconeogenesis and glycogenolysis by FGF21 in zebrafish might be occurring by the mediation of this hormone. Interestingly, our results suggest that not only gluconeogenic but also glycolytic pathways appear to be activated by FGF21 in the zebrafish liver, as suggested by the increased expression and activity of the glycolytic enzymes Gck, PfkA, PfkB and Pk. While these two processes are normally regulated so that they do not occur at the same time, previous reports have described the simultaneous stimulation of gluconeogenesis and glycolysis in fish^40^. In the context of the present study, it is possible that the high amount of glucose that might enter into the hepatic cells or is being synthesized in response to FGF21 is at the same time hydrolyzed to produce pyruvate. Resulting pyruvate can enter into the Krebs cycle and be used for obtaining energy. Indeed, FGF21 was previously observed to increase the Krebs cycle flux in mice^13^. A tendency towards increasing the gain of energy in response to FGF21 is also supported by the FGF21-induced upregulation of mRNAs encoding ATP5f1b, a subunit of mitochondrial ATP synthase.

In the Krebs cycle, pyruvate is first converted into citrate, which apart from being used in that cycle, could also be released into the cytoplasm through a specific mitochondrial carrier and be used as a substrate for fatty acid biosynthesis^41^. Our results point towards the possibility that FGF21 increases lipogenesis in the zebrafish liver, as suggested by the increased expression and/or activity of the enzymes Acly (catalyzes conversion of citrate into acetyl-CoA), Acc (catalyzes conversion of acetyl-coA into malonyl-CoA) and Fas (catalyzes conversion of malonyl-CoA into palmitate). Palmitate formed by the action of Fas can be either β-oxidized in order to obtain more energy or incorporated into phospholipids. In the present study, the unaltered expression of *acadm* and *echs1 in vivo* and *in vitro*, and the reduced expression (*in vitro*) and activity of Hoad (all enzymes involved in β-oxidation) in response to FGF21, seem to indicate that β-oxidation is not being upregulated by this peptide in the zebrafish liver. Instead, palmitate might be used for synthesizing phospholipids for maintaining cell membrane structure. In mammals, it has been described that about 60–70% of palmitate in the liver and most other tissues is incorporated into phospholipids, and only 20–30% is β-oxidized^42^. The observations that FGF21 seems to promote lipogenesis and does not stimulate β-oxidation in the zebrafish liver are opposite to that reported for mammals^11,13,14^. Additionally, the latter does not agree with the increased expression and activity of Cpt1a, which allows the entrance of fatty acids into the mitochondria. This, however, could be related to an increase in ketogenesis by FGF21, which indeed is suggested by the increased expression of *hmgcl* and *acat1* in response to treatment with the peptide. Ketogenesis was reported to be induced by FGF21 in the mammalian liver^11,12^. This might indicate a need to produce extra energy when glycogen stores are depleted and glucose levels are low, a state that occurs during prolonged periods of fasting. The increase in Acly expression and activity supports the hypothesis that FGF21 might be stimulating ketogenesis in the zebrafish liver, given that the acetyl-CoA produced in the reaction catalyzed Acly can be used for synthesizing ketone bodies. A limitation of the present research is that it studied only the gene expression and activity of key metabolic enzymes, but not the cellular metabolism. While the observed effects of FGF21 on the metabolic machinery point to an alteration of the metabolic processes discussed, further studies on metabolite levels should be needed to confirm such a role for FGF21 in fish. In addition, the liver samples from *in vivo* studies are modulated by an array of hormones and metabolites that represent a multiple, redundant milieu. Meanwhile, the results obtained from ZFL cells reflect a direct action of FGF21, without the influence of the *in vivo* milieu. Therefore, direct comparison of results obtained using these two models has its limitations.

The last aim of this research was to evaluate whether Ppargc1*α* and Pparα mediate the actions of FGF21 in the zebrafish liver. PPARGC1*α* and PPARα are key transcriptional regulators of energy homeostasis. Thus, the induction of PPARGC1*α* allows this protein to coactivate several transcription factors, including PPARα, which in turn regulates the expression of key genes and enzymes involved in gluconeogenesis, fatty acid oxidation, and other metabolic processes^43^. In mammals, FGF21 has been identified as a downstream of PPARα activation^11,12,35,44^, but also as a regulator of PPARGC1*α*^13,31,37,45^, suggesting that the mechanism of action for its physiological effects is mediated at least by this coactivator. The adipokine adiponectin has also been reported to mediate the metabolic effects of FGF21 in the mice liver^46^. In the present study, we reported that FGF21 modulates the expression of *ppargc1α in vitro*, suggesting a similar mechanism of action for FGF21 in fish than in mammals. However, the lack of FGF21-induced effects on *ppargc1α* mRNAs *in vivo* needs this hypothesis to be further confirmed. We also reported that Pparα might be a mediator of FGF21 actions in zebrafish, as its mRNA levels resulted modulated in ZFL cells treated by the peptide.

In summary, this study characterized FGF21 as an important orexigen in zebrafish, and reported that the expression of mRNAs encoding the peptide as well as its receptors is importantly modulated by feeding and food deprivation in central and peripheral tissues. We also showed for the first time in fish that FGF21 exerts an important role in regulating glucose and lipid metabolism in the zebrafish liver. Specifically, we observed that FGF21 appears to stimulate a fasting metabolic state, as it has been reported in mammals. Results presented here add significant new information to our growing knowledge on naturally occurring regulators of vertebrate physiology. To study the putative regulation of the FGF21 system by the composition of the diet, whether metabolic roles of FGF21 differ in the fed and fasted state, and a deeper characterization of the mechanisms underlying the physiological actions of the peptide, are important new directions to consider for future research.

## Methods

### Animal models

Zebrafish (*Danio rerio*; body weight (bw): ~1 g) were obtained from the Aquatic Toxicology Research Facility at the University of Saskatchewan and housed in 10 L aquaria with a constant flow of temperature-controlled water (26 ± 1 °C). Fish were maintained under a 12 h light:12 h darkness (12 L:12D) photoperiod (lights on at 07:00 h), and fed daily at 11:00 h with commercial slow-sinking pellets (1% bw; Aqueon Catalog # 06053, Franklin, WI, USA). All fish studies adhered to the Canadian Council of Animal Care guidelines, and research protocols were approved by the Animal Research Ethics Board of the University of Saskatchewan (Protocol Number 2012–0082).

### ZFL cells

Zebrafish liver (ZFL) cells were purchased from ATCC (Catalog # ATCC^®^ CRL-2643^™^; Manassas, VA, USA) and cultured at 28 °C under a 100% air atmosphere. Complete culture media consisted of 50% Leibovitz’s L-15 (Thermo Fisher Scientific, Waltham, MA, USA), 35% Dulbecco’s Modified Eagle Medium (DMEM) High Glucose (Sigma-Aldrich, Oakville, ON, Canada) and 15% Ham’s F12 (ATCC) (all without sodium bicarbonate), supplemented with 0.15 g/L sodium bicarbonate, 15 mM HEPES, 0.01 mg/mL bovine insulin, 50 ng/mL mouse epidermal growth factor (EGF), 5% heat-inactivated fetal bovine serum and 0.5% trout serum. At 80% confluency, ZFL cells were seeded at 5 × 10^5^ cells/well in 24-well plates or 1 × 10^6^ cells/well in 6-well plates, and the studies were performed when cells were 80–90% confluent (typically 48–72 h after seeding). An additional batch of cells was seeded in chamber slides for immunocytochemistry.

### Reagents

The human recombinant FGF21 protein (amino acids 29 to 209) was obtained from Abcam (Catalog # ab54141; Toronto, ON, Canada). Recombinant human FGF21 was previously used in studies using fish models and was reported to exert biological effects^27^. For *in vivo* studies, peptide was prepared in sterile saline (0.9% NaCl) at a concentration of 1, 10 or 100 ng/10 µL saline. For *in vitro* studies, FGF21 was diluted in ZFL complete culture media at concentrations of 0.1 nM, 1 nM and 10 nM.

### Experimental designs

#### Distribution of the FGF21 system mRNAs in zebrafish tissues involved in feeding and metabolism

Six zebrafish were anesthetized using tricaine methanesulfonate (MS-222; Syndel Laboratories, Nanaimo, BC, Canada) and sacrificed by decapitation. Samples of brain (without the hypothalamus), hypothalamus, foregut (intestinal bulb and anterior portion of the intestine), hindgut (posterior portion of the intestine) and liver (n = 6) were collected, immediately frozen in liquid nitrogen and stored at −80 °C until quantification of gene expression (see *Real-time quantitative PCR* section).

#### Effects of FGF21 on food intake

A total number of 12 tanks (3 zebrafish/tank) were set up, where fish were maintained as described earlier. Following a 3-week acclimation period, fish were divided into four experimental groups (3 tanks/group): i) Control, ii) 1 ng/g bw FGF21, iii) 10 ng/g bw FGF21, and iv) 100 ng/g bw FGF21. Food intake was registered daily during one week before treatment to evaluate basal levels of food intake. For this, a pre-weighed amount of food was offered to fish in each tank. After feeding for 30 min, the uneaten food was collected, dried for 24 h and weighed. The amount of food consumed by all fish in each tank was calculated by subtracting the dry weight of the amount of food retrieved from the tank after 30 min of feeding from the dry weight of the total amount of food originally provided. On the day of experiment, fish were lightly anesthetized with MS-222 at the scheduled feeding time, weighed and IP injected with either saline alone (control group) or containing 1, 10 or 100 ng/g of FGF21. Immediately after the injections, fish were allowed to recover (5 min) and were offered a pre-weighed quantity of food. Uneaten food was recovered 1 h post-injections, dried and weighed in order to calculate the amount of food intake. Fish were again fed a pre-weighed amount of food at 2, 6 and 24 h post-injections, and the amount of food ingested at each time was quantified as described above. The experiment was repeated three times, and results shown correspond to the mean of three experiments.

#### Effects of fasting on the expression of the FGF21 system

Fish were divided into two groups, control and experimental (n = 6/group). Following a 2-week acclimation period, fish of the experimental group were not provided food for 7 days, while the fish in the control group were fed daily. On day 7, control and fasted fish were sacrificed at 11:30 h, and samples of whole brain, foregut and liver were collected as above described. The expression of FGF21 system genes was quantified as described below (see *Real-time quantitative PCR* section).

#### Periprandial changes in the expression of the FGF21 system

Seven groups of fish (n = 6/group) were established and acclimated to tank conditions during 2 weeks. On the day of the experiment, fish from three aquaria (6 fish per sampling time) were sampled at −3 h, −1 h and 0 h before the scheduled feeding time. At the scheduled feeding time, two of the remaining four tanks were fed while food was withheld from the other two tanks. Fish from both fed and unfed tanks were sampled at +1 h and +3 h after scheduled feeding time. Samples of brain, foregut and liver were collected as above described until analysis of mRNA abundance (see *Real-time quantitative PCR* section).

### *In vivo* effects of FGF21 on the expression of genes involved in appetite regulation and glucose and lipid metabolism

Fish were divided into four experimental groups (6 fish/group): i) Control, ii) 1 ng/g bw FGF21, iii) 10 ng/g bw FGF21, and iv) 100 ng/g bw FGF21. After a 2-week acclimation period, fish were anesthetized at the scheduled feeding time and IP injected with either saline alone (control group) or containing 1, 10 or 100 ng/g of FGF21. Subsequently, fish were allowed to recover and fed. Following 2 h, fish were anesthetized again and sacrificed by decapitation in order to collect samples of brain, foregut and liver. Samples were kept at −80 °C until mRNA quantification (see *Real-time quantitative PCR* section).

### *In vitro* concentration- and time- dependent effects of FGF21 on the expression of genes involved in glucose and lipid metabolism

ZFL cells were seeded at 5 × 10^5^ cells/well in 24-well plates and grown to confluency as described earlier. Then, culture media was replaced by 1 mL of fresh media alone (6 wells) or containing 0.1, 1 or 10 nM FGF21 (6 wells each), and plates were incubated for 1 h and 6 h. At the end of each culture time, media was removed and 500 µL of PureZOL^TM^ RNA Isolation Reagent (Bio-Rad, Mississauga, ON, Canada) was added to each well. Cells were then scraped from the bottom of the wells, collected and frozen at −80 °C until total RNA was extracted (see *Real-time quantitative PCR* section). This experiment was repeated twice.

### *In vitro* effects of FGF21 on glucose transporters levels and the activity of enzymes implicated in glucose and lipid metabolism

For this assay, we chose the concentrations and time in which FGF21 exerts the most significant inductions in mRNA expression. ZFL cells were seeded at 1 × 10^6^ cells/well in 6-well plates and grown to confluency. Once 80–90% confluency was achieved, media was replaced by 1 mL of fresh media alone (4 wells for Western blot and 8 wells for assessment of enzymatic activity) or containing 1 or 10 nM FGF21 (4 wells each for Western blot and 8 wells each for assessment of enzymatic activity). After an incubation period of 1 h, media was removed and 300 µL of lysis buffer was added to each well. For Western blot analysis, lysis buffer consisted of T-PER Tissue Protein Extraction Reagent (Thermo Fisher Scientific, Waltham, MA, USA), while samples for enzymatic activity assessment were lysed in a 80 mM Trizma buffer (pH 7.6) containing 5 mM EDTA, 2.6 mM DTT and protease inhibitor cocktail (Thermo Fisher Scientific). After the addition of the buffer, cells were scraped, transferred to tubes and stored at −80 °C until further analysis (see Sections *Western blot* and *Enzymatic activity assessment*).

### Real-time quantitative PCR

Total RNA was extracted using PureZOL^TM^ RNA Isolation Reagent (Bio-Rad). RNA purity was checked by optical density (OD) absorption ratio (OD 260 nm/OD 280 nm) using a NanoDrop 2000c (Thermo, Vantaa, Finland). Synthesis of cDNAs from 1 µg of total RNA was conducted using iScript Reverse Transcription Supermix for RT-qPCR as directed by the manufacturer (BioRad, Canada). The first strand cDNA fragments obtained were used as template to amplify the target and reference genes using specific forward (Fw) and reverse (Rv) primers: *β-actin* (Fw 5′-TTCAAACGAACGACCAACCT-3′ and Rv 5′-TTCCGCATCCTGAGTCAATG-3′), *acaca* (Fw 5′-AATCAGGTGGTACGGATGGC-3′ and Rv 5′-GGATGTTCCCTCTGTTGGGG-3′), *acadm* (Fw 5′-AAGGTTTTGAGGGCAGGTGT-3′ and Rv 5′-ACTCTTTCTGCTGCTCGGTT-3′), *acat1* (Fw 5′-ATCCCGCAGAGAGGAAAACC-3′ and Rv 5′-CGAGTGGTGTGACGTTGAGT-3′), *acly* (Fw 5′-CCCACACCGCTAACTTCCTT-3′ and Rv 5′-TCCTGGCGCGAACAAACATA-3′), *agrp* (Fw 5′-CTGGGACGTGAGCACTACAG-3′ and Rv 5′-AAGGTGCTCCATTTCAGGCA-3′), *atp5f1b* (Fw 5′-ATGATGTAGCCCGTGGTGTG-3′ and Rv 5′-GCCCAAGTGTCCAGTGAAGA-3′), *cart* (Fw 5′-GAGAGACTTGGCTGAGGCAC-3′ and Rv 5′-GAAAGTGTTGCAGGCGGTTC-3′), *cck* (Fw 5′-ACGCTGGACTCTGTGTAT-3′ and Rv 5′-CTTCATCGTCCTCTGGTTTG-3′), *cpt1a* (Fw 5′-TCTACCTGAGAGGTCGTGGG-3′ and Rv 5′-TAGCCGTTCCCATTGAGCAG-3′), *echs1* (Fw 5′-CCCTTGCGATGGAGATGGTT-3′ and Rv 5′-CGACTGCCCTCAGCTAAAGT-3′), *fasn* (Fw 5′-GGCGAGTGGTCAGTCAGTTA-3′ and Rv 5′-TTGTTCGACTCAGGAAGGCG-3′), *fbp1a* (Fw 5′-TGGCGAGTTCATTCTGGTGG-3′ and Rv 5′-TCTGCCACCATTGAGCCTAC-3′), *fbp1b* (Fw 5′-GAGTCCCAAGGGCAAGCTAA-3′ and Rv 5′-TACAGGAACCCTCTGGTGGA-3′) *fgf21* (Fw 5′-CGGCAATCCTCTTTCTCGCT-3′ and Rv 5′-TAGTGGGTCGTCAGAGTCCA-3′), *fgfr1a* (Fw 5′-GCGGAATCTCGCTGTATCT-3′ and Rv 5′-TCGGTCGGCAGATCAAAACA-3′), *fgfr1b* (Fw 5′-TGACCGATACCACCTTCCCT-3′ and Rv 5′-CGGTTGGTTTAGTGGTTGCG-3′), *fgfr2b* (Fw 5′-GTGGAAGTCCTCATGCTCACC-3′ and Rv 5′-CAACAGGAATTGTTGGCCTCA-3′), *fgfr2c* (Fw 5′-GGATAAAGAGATCGAGGTGCTCTAC-3′ and Rv 5′-ACCAAGCAGTGTGATAGGAGATC-3′), *fgfr3* (Fw 5′-CGTTCGAGCAGTATTCACCG-3′ and Rv 5′-CAGTGCAGCCAAACCCTAAAG-3′), *fgfr4* (Fw 5′-TTTCGGGGTTTTGATGTGGGA-3′ and Rv 5′-ATTGACACCAGCACCCTATCG-3′), *g6pcb* (Fw 5′-CATCTGGACACCACACCCTT-3′ and Rv 5′-TGGGTGGTCTGAACGAGTCT-3′), *gck* (Fw 5′-GACACAGGGGACAGAAAGCA-3′ and Rv 5′-CCACCCCCACAGTGATCTTT-3′), *glut2* (Fw 5′-GGATACAGCTTGGGCGTCAT-3′ and Rv 5′-CTCTGTGCCATTTCCCCCTT-3′), *glycogen phosphorylase* (Fw 5′-TGTAAAGTCCTCGCGCACA-3′ and Rv 5′-ACATCCCCGAGTCCTGCTAT-3′), *grl* (Fw 5′-ATGGTCCCGTGCTTCAGAAT-3′ and Rv 5′-TGCACCCACTTTGCTACAGA-3′), *hadh* (Fw 5′-TGGAGGCTGTTCGGTTACAC-3′ and Rv 5′-TTTGTTGAGCAGGGGACTGG-3′), *hmgcl* (Fw 5′-AGCCACGTCAATAGAAAGCAGT-3′ and Rv 5′-CCTGGTCCTTTTGCCTCTGT-3′), *leptin a* (Fw 5′-GCTCTCCGCTCAACCTGTATT-3′ and Rv 5′-TTTGCCCGTCAATGTGTTCC-3′), *leptin b* (Fw 5′-TTCCCCGTCACCTCCAACTA-3′ and Rv 5′-CCTTGCATGTGCCATTGTGTT-3′), *npy* (Fw 5′-GGCCACCAGATCTCATAAA-3′ and Rv 5′-GCGCACATTGACGTATTT-3′), *nucb2 a* (Fw 5′-AGGAGCGGCATGAAGAATTT-3′ and Rv 5′-GATGGTTGACTTTGGGGTGA-3′), *nucb2 b* (Fw 5′-TCTGTGGGCTTGTTTGGATG-3′ and Rv 5′-TTCTCTCTGAAATGCGGGTC-3′), *pck1* (Fw 5′-AGCTCTTCAGGGTCTCGCA-3′ and Rv 5′-TAACGTGTGTGTTGCGTGTCTT-3′), *pck2* (Fw 5′-TCCTTCGGCAGTGGTTATGG-3′ and Rv 5′-GCTGCTGCAATGTACCGTTT-3′), *pfkla* (Fw 5′-AGGTATGAACGCAGCCATCC-3′ and Rv 5′-TGCCAATCACTGTTCCTCCC-3′), *pfklb* (Fw 5′-TTTGAGCACAGGATGCCGAA-3′ and Rv 5′-TCGATGCTAAGGGTTCGACG-3′), *pklr* (Fw 5′-CCAGTTTAACACGCGCGGC-3′ and Rv 5′-GGGAAGTGTCCTTTGGCTGT-3′), *pomc* (Fw 5′-CACTGCTCACACTCTTCA-3′ and Rv 5′-GCCCACCTTCGTTTCTAT-3′), *pparα a* (Fw 5′-TAATCCACTCTCTGCGGCTC-3′ and Rv 5′-CATGTTACTGCCGGTCTCCT-3′), *ppargc1α* (Fw 5′-AAAGCCGGTGAAGCCAAGAG-3′ and Rv 5′-GGTCACTGCAACACAGAGGA-3′), *sglt1* (Fw 5′-TGTCCGTCATGTTGGCTTCA-3′ and Rv 5′-TCTGAGCCGTCTGAACGATG-3′), all obtained from IDT (Toronto, ON, Canada). Real-time quantitative PCRs (RT-qPCRs) were performed in 96-well plate loaded with 1 µL of cDNA and 500 nM of each forward and reverse primer in a final volume of 10 µL. Supermix used was SensiFAST SYBR No-ROX Kit (FroggaBio, Toronto, ON, Canada). Each PCR run included a standard curve for the corresponding gene made of two replicates of four serial dilution points, and water instead of cDNA as control in order to ensure that the reagents were not contaminated. RT-qPCR cycling conditions consisted of an initial step of 95 °C for 3 min, and 35 cycles of 95 °C for 10 sec and 60 °C for 25 sec. A melting curve was systematically monitored (temperature gradient at 0.5 °C/5 sec from 65 to 95 °C) at the end of each run to confirm specificity of the amplification reaction. All runs were performed using a CFX Connect Real-Time System (Bio-Rad). The 2−ΔΔCt method^47^ was used to determine the relative mRNA expression.

### Western blot

Protein extraction and quantification, and Western blot protocol were performed as previously described^48^. Briefly, the samples (containing 20 µg protein) were boiled, electrophoresed and transferred to a nitrocellulose membrane (Bio-Rad). After blocking using 1X RapidBlock solution (AMRESCO, Toronto, ON, Canada), target proteins within the membrane were detected by overnight incubation with specific primary antibody: goat polyclonal to GLUT2 (1:500 dilution; Catalog # ab111117; Abcam) and rabbit polyclonal to SGLT1 (1:500 dilution; Catalog # ab14686; Abcam). Vinculin protein was used for normalization and was detected using rabbit antiserum directed against mouse vinculin (1:1000 dilution; Catalog # ab129002, Abcam). As secondary antibodies, goat anti-rabbit or rabbit anti-goat IgG (H + L) HRP conjugate (Bio-Rad) diluted 1:2000 were used. Protein were visualized using Clarity™ Western ECL substrate (Bio-Rad) in a ChemiDoc™ MP imaging system (Bio-Rad) with chemiluminescence detection. Blot images were analysed using ImageLab software and band density of vinculin was used to normalize glucose transporter protein density.

### Immunocytochemistry

Slide chambers seeded with ZFL cells were used for immunocytochemical localization of Glut2 and Sglt1 within the cells. At 80–90% confluency, media was removed from the chambers and chambers were washed twice in 400 µL of PBS. Cells were then fixed by incubation with 400 µL of 4% paraformaldehyde solution during 20 min at room temperature. After fixation, wells were washed twice in 400 µL of PBS and twice in 400 µL of wash buffer (0.1% BSA in 1X PBS), and blocked in 0.1 M PBS containing 0.5% of bovine serum albumin for 45 min before being incubated overnight with primary antibodies, each diluted 1:200, at room temperature. Primary antibodies used were: goat polyclonal to GLUT2 (Catalog # ab111117) and rabbit polyclonal to SGLT1 (Catalog # ab14686), both from Abcam. Since heterologous antibodies were used here, it is likely that a certain degree of non-specificity exists in our findings. Therefore, the suffix “-like” was used to refer to immunostaining obtained. The following day, wells were washed two times in wash buffer, and 400 µL of a solution containing the corresponding secondary antibodies (donkey anti-goat IgG Alexa Fluor 488 or goat anti-rabbit IgG Alexa Fluor 488, dilution 1:2000; Invitrogen, Burlington, ON, Canada) were added. A separate set of negative control slides, where primary antibodies were omitted and only the secondary antibodies were used, were included. All primary and secondary antibodies were diluted in an antibody diluent (Dako, Mississauga, ON, Canada). After final wash in wash buffer, chambers were removed and slides were mounted using VECTASHIELD mounting medium containing 4′,6-diamidino-2-phenylindole (DAPI; Vector Laboratories, Burlington, ON, Canada). Slides were then assessed using a Nikon Eclipse Ti-Inverted fluorescence microscope (Nikon Instruments, Melville, NY, USA), and images were captured using a Nikon DS-Qi1 MC camera. Images were analysed using the NiS Elements Basic Research Imaging Software on a Lenovo ThinkPad workstation. Micrographs were generated in the “TIFF” format and adjusted linearly for light and contrast using Photoshop CS6 (Adobe Systems Inc., San Jose, CA, USA).

### Enzymatic activity assessment

Enzymatic activities were determined following protocols previously described by Soengas and coworkers for fish^49,50^, slightly adapted to their use in ZFL cells. Briefly, enzymatic reactions were assessed in 96-well plates loaded with ZFL homogenates (10–50 μL) and the corresponding reaction buffer (omitting the substrate in control wells), and allowing the reactions to proceed at 37 °C for pre-established times (3–25 min). Reaction buffers used for the enzymes tested are as follows. Gck (Enzyme Commission, EC, number 2.7.1.2) buffer consisted in a Trizma buffer (80 mM, pH 8.0) containing 10.2 mM KCl, 37.5 mM MgCl_2_, 11.5 mM KH_2_PO_4_, 20 mM NaHCO_3_, 4 mM EDTA, 2.6 mM DTT, 2 mM NADP^+^, 7 mM ATP, 0.13 U mL^−1^ glucose 6-phosphate dehydrogenase, 0.13 U mL^−1^ 6-phosphogluconate dehydrogenase, and 1.2 M and 20 mM D-glucose (omitted for controls). Pk (EC 2.7.1.40) was assessed in an imidazole buffer (50 mM, pH 7.4) containing 100 mM KCl, 10 mM MgCl_2_, 0.5 mM ADP, 0.15 mM NADH, 22 U mL^−1^ lactate dehydrogenase, and 1.5 mM phosphoenolpyruvate (omitted for controls). Pepck (EC 4.1.1.32) was assessed in a Trizma buffer (50 mM, pH 7.5) containing 1 mM MnCl_2_, 20 mM NaHCO_3_, 1.5 mM phosphoenolpyruvate, 0.3 mM NADH, 1.8 U mL^−1^ malate dehydrogenase, and 0.5 mM 2′-deoxyguanosine-5-diphosphate (omitted for controls). Acly (EC 4.1.3.8) was assessed in a Trizma buffer (50 mM, pH 7.8) containing 100 mM KCl, 10 mM MgCl_2_, 20 mM citric acid, 10 mM β-mercaptoethanol, 5 mM ATP, 0.3 mM NADH, 7.4 U mL^−1^ malate dehydrogenase, and 500 μM Coenzyme A (omitted for controls). Fas (EC 2.3.1.85) was determined in a phosphate buffer (0.1 mM K_2_HPO_4_ and 0.1 mM KH_2_PO_4_, pH 6.5) containing 0.1 mM NADPH, 25 μM acetyl-CoA, and 100 μM malonyl-CoA (omitted for controls). Cpt1a (EC 2.3.1.21) was assessed in a Trizma buffer (75 mM, pH 8.0) containing 1.5 mM EDTA, 0.25 mM 5,5′-dithiobis(2-nitrobenzoic acid) (DTNB), 50 μM palmitoyl-CoA, and 2 mM l-carnitine (omitted for controls). Finally, Hoad (EC 1.1.1.35) activity was assessed in an imidazole buffer (50 mM, pH 7.6) containing 0.15 mM NADH, and 300 μM acetoacetyl-CoA (omitted for controls). Reaction rates of enzymes were determined by the decrease in absorbance of NADH at 340 nm (in the case of Pk, Pepck, Acly, Fas and Hoad), the increase in absorbance of NADPH at 340 nm (Gk), or the increase of DTNB-CoA complex at 412 nm (Cpt1a). All measurements were carried out in a SpectraMax 190 microplate reader (Molecular Devices, San Jose, CA, USA). Enzyme activities are expressed per mg protein, which was assayed by Bradford assay (Bio-Rad).

### Statistics

Statistical differences between groups were assessed using either Student′s t-test (for comparisons between two groups) or one-way ANOVA followed by Student-Newman-Keuls multiple comparison test (for comparisons among multiple groups), after data were checked for normality and homogeneity of variance. Data that failed one of these requirements were log-transformed and re-checked. Significance was assigned when p < 0.05. All analyses were carried out using SigmaPlot version 12.0 (Systat Software Inc., San Jose, CA, USA) statistics package.

## Supplementary information

Supplementary figures.
