# Supplementary material for: FGF21 Mimics a Fasting-Induced Metabolic State and Increases Appetite in Zebrafish
Source: Sci Rep. 2020 Apr 24;10:6993. doi: 10.1038/s41598-020-63726-w (PMC7181725; doi:10.1038/s41598-020-63726-w)

FGF21 Mimics a Fasting-Induced Metabolic State and Increases Appetite in Zebrafish

Ayelén Melisa Blanco[1,2], Juan Ignacio Bertucci[1,3], Suraj Unniappan[1*]

[1] Laboratory of Integrative Neuroendocrinology, Department of Veterinary Biomedical Sciences, Western College of Veterinary Medicine, University of Saskatchewan, Saskatoon, Saskatchewan, Canada.

[2] Laboratorio de Fisioloxía Animal, Departamento de Bioloxía Funcional e Ciencias da Saúde, Facultade de Bioloxía and Centro de Investigación Mariña, Universidade de Vigo, Vigo, Pontevedra, Spain.

[3] Toxicology Centre, University of Saskatchewan, Saskatoon, Saskatchewan, Canada.

Supplementary Figure 1. Full-length images of immunoblots included in Figures 5f and i. Bands shown in Figures 5f and i are boxed. For each protein, two separate blots containing two samples per treatment were performed. Figure here shows one of those two blots. **(a)** GLUT2. **(b)** SGLT1. Molecular markers are included. See corresponding figure legend for details.

a

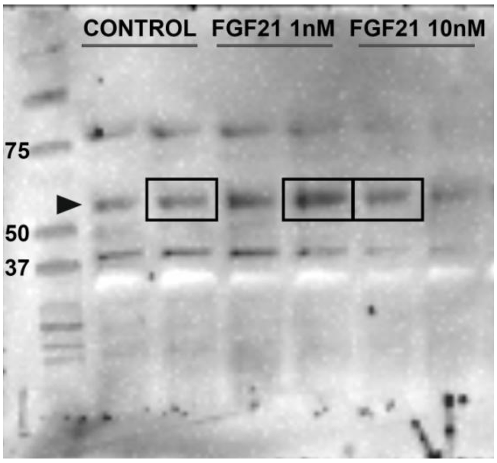

b

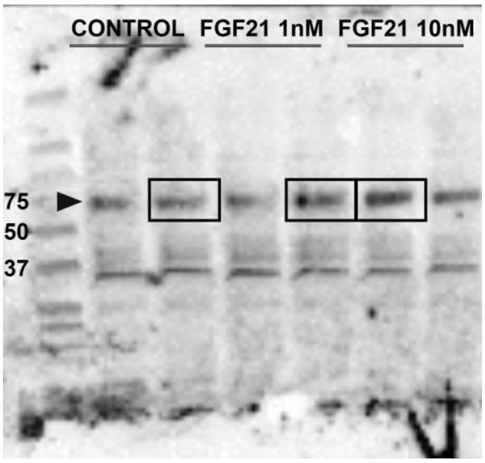

Supplementary Figure 2. Aligment between zebrafish (GenBank Accesion number: NP_001038789.1) and human (GenBank Accesion number: AAQ89444.1) FGF21 proteins. Identical amino acids between the two sequences are color-shaded. The conserved cysteine residue is boxed.

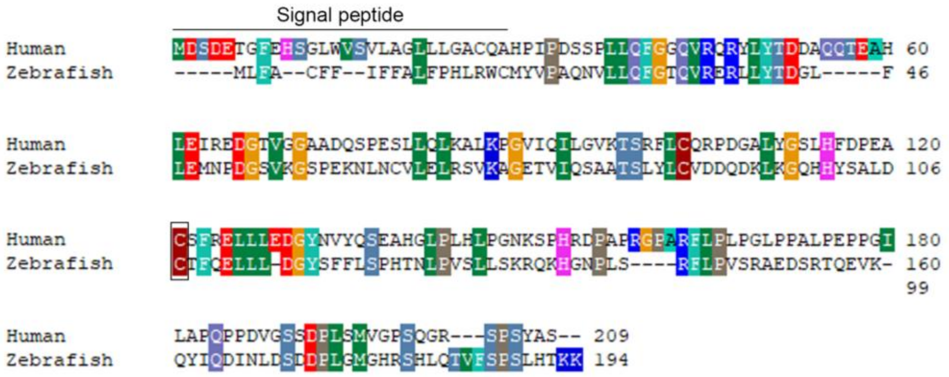

Supplement: Supplementary file 1 — Supplementary figures. [file 41598_2020_63726_MOESM1_ESM.pdf]
